# Parathyroid hormone increases alveolar bone homoeostasis during orthodontic tooth movement in rats with periodontitis via crosstalk between STAT3 and β-catenin

**DOI:** 10.1038/s41368-020-00104-2

**Published:** 2020-12-30

**Authors:** Cheng Zhang, Tiancheng Li, Chenchen Zhou, Li Huang, Yuyu Li, Han Wang, Peipei Duan, Shujuan Zou, Li Mei

**Affiliations:** 1 State Key Laboratory of Oral Diseases & National Clinical Research Center for Oral Diseases & Department of Orthodontics, West China Hospital of Stomatology, Sichuan University, Chengdu, China; 2 Discipline of Orthodontics, Faculty of Dentistry, Department of Oral Sciences, Sir John Walsh Research Institute, University of Otago, Otago, New Zealand

**Keywords:** Bone, Hormone receptors

## Abstract

Periodontitis patients are at risk of alveolar bone loss during orthodontic treatment. The aim of this study was to investigate whether intermittent parathyroid hormone (1–34) treatment (iPTH) could reduce alveolar bone loss during orthodontic tooth movement (OTM) in individuals with periodontitis and the underlying mechanism. A rat model of OTM in the context of periodontitis was established and alveolar bone loss was observed. The control, iPTH and iPTH + stattic groups received injections of vehicle, PTH and vehicle, or PTH and the signal transducer and activator of transcription 3 (STAT3) inhibitor stattic, respectively. iPTH prevented alveolar bone loss by enhancing osteogenesis and suppressing bone resorption in the alveolar bone during OTM in rats with periodontitis. This effect of iPTH was along with STAT3 activation and reduced by a local injection of stattic. iPTH promoted osteoblastic differentiation and might further regulate the Wnt/β-catenin pathway in a STAT3-dependent manner. The findings of this study suggest that iPTH might reduce alveolar bone loss during OTM in rats with periodontitis through STAT3/β-catenin crosstalk.

## Introduction

There is an increasing number of periodontal patients seeking orthodontic treatment for improvements in function and aesthetics.^1^ It has been found that appropriate orthodontic-periodontal combination therapies can improve periodontal conditions.^2,3^ However, in patients with periodontitis (PD), there can be an increased risk of alveolar bone loss due to unfavourable orthodontic conditions, e.g., poor oral hygiene due to increased difficulty in tooth brushing and formation of biofilms^4^ with higher periodontal pathogenicity leads to the recurrence of active PD and exacerbation of alveolar bone loss.^5^ In addition, orthodontic force itself can induce an inflammatory response, which may amplify the level of inflammation in the periodontium and result in elevated bone resorption.^6–8^ The situation may be even worse if a patient smokes or suffers from diabetes, immune disorders, osteoporosis and genetic predisposition.^9–12^ Thus, it is of great importance to prevent alveolar bone loss during orthodontic treatment in patients with PD.

Currently, intermittent parathyroid hormone (1–34) treatment (iPTH) has been used for treating postmenopausal osteoporosis and iPTH also shows promising potential for regenerating alveolar bone and preventing alveolar bone loss in rats.^13–18^ Clinical studies have shown the effectiveness of iPTH in improving periodontal indices in humans.^19,20^ Therefore, iPTH may be a pharmaceutical approach to prevent alveolar bone loss during orthodontic treatment in PD patients. The anabolic effect of iPTH relies on promoting osteogenesis and increasing osteoblastic cell numbers.^21^ Several signalling pathways and cytokines have been proven to be involved in the mechanism by which iPTH regulates bone homoeostasis. Activation of cAMP/PKA^22^ and Wnt/β-catenin signalling^23,24^ plays a critical role in this process. Other PTH-responsive factors, such as the Notch ligand Jagged-1,^25,26^ c-fos,^27,28^ insulin-like growth factor-1,^29,30^ fibroblast growth factor 2^31^ and interleukin (IL)-6 family cytokines,^32,33^ also contribute to the anabolic effect of PTH on bone homoeostasis.

Members of the IL-6 family of cytokines bind to their coreceptor glycoprotein 130 and thus activate Janus kinases (JAKs) and the downstream signal transducer and activator of transcription 3 (STAT3). In addition, the IL-10 and fibroblast growth factor-23 levels are increased by iPTH stimulation and may also trigger STAT3 activation.^34,35^ STAT3 is involved in bone metabolism and many other biological processes. Loss-of-function mutation of STAT3 can induce hyperimmunoglobulin G syndrome, which is characterized by symptoms of osteoporosis, recurrent minimal bone fracture and skeletal morphological defects.^36^ Osteoblast/osteocyte-specific STAT3-knockout mice exhibit significantly lower bone mass due to reduced bone formation and enhanced bone resorption.^37^ However, whether STAT3 is responsible for the anabolic effect of iPTH remains unknown.

The crosstalk between the JAK/STAT pathway and Wnt/β-catenin pathway has been found to play important roles in the processes of proliferation, apoptosis and angiogenesis.^38^ Research has shown that STAT3 and β-catenin directly interact in the nucleus,^39^ and STAT3 positively regulates the transcriptional activity of β-catenin.^40^ Thus, we hypothesized that the interaction between STAT3 and Wnt/β-catenin signalling may also participate in the mechanism of action of iPTH.

This study aimed to investigate the effect of iPTH on alveolar bone homoeostasis during orthodontic tooth movement (OTM) in individuals with PD, as well as the underlying molecular mechanism, including STAT3 and Wnt/β-catenin.

## Results

### OTM exacerbated alveolar bone loss in individuals with PD

**Fig. 1:**
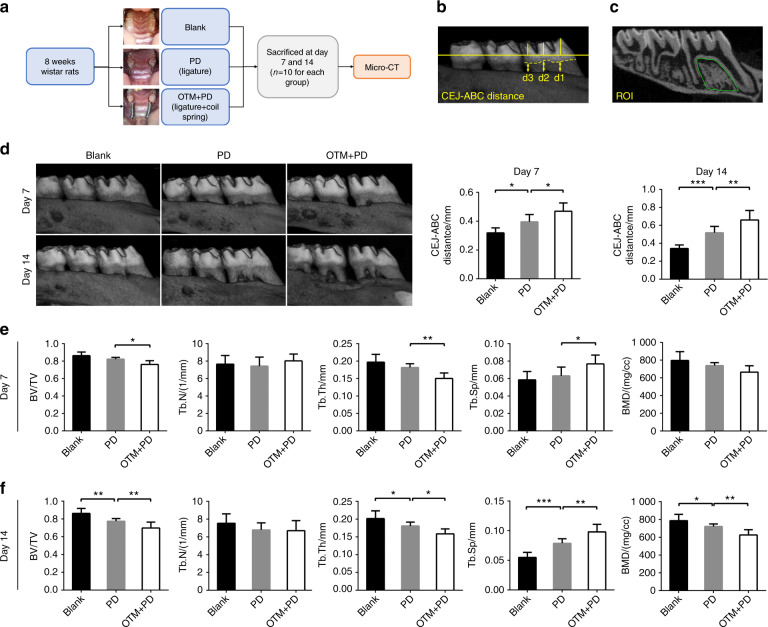
Micro-CT showed that OTM + PD caused increased alveolar bone loss. **a** A flowchart of the in vivo study Part I. **b** Methods for measuring the CEJ-ABC distance. **c** ROI for trabecular morphometry analysis. **d** Buccal images of specimens showing the vertical bone height in the blank, PD and OTM + PD groups and quantitative analysis of the CEJ-ABC distance. **e**, **f** BV/TV, Tb. N, Tb. Th, Tb. Sp and BMD in each group at days 7 and 14, respectively. *P < 0.05, **P < 0.01, ***P < 0.001, ****P < 0.000 1 by one-way ANOVA with Tukey’s post hoc test

A model of OTM in the context of PD was established in rats, and alveolar bone loss in this model was verified (Fig. 1a–c). On days 7 and 14, the PD group showed a significant increase in the cementoenamel junction-alveolar bone crest (CEJ-ABC) distance compared with the blank group (Fig. 1d). On day 14, in the PD group, the bone volume fraction (BV/TV), trabecular thickness (Tb. Th) and bone mineral density (BMD) were significantly lower and the trabecular separation (Tb. Sp) was significantly higher than those in the blank group (Fig. 1f).

Compared with that in the PD group, the CEJ-ABC distance in the OTM + PD group was further exacerbated on day 7 and 14 (Fig. 1d). In the OTM + PD group, the BV/TV and Tb. Th were significantly lower, whereas the Tb. Sp was notably higher than those in the PD group on day 7 and 14. The BMD of the OTM + PD group was significantly lower than that of the PD group on day 14 (Fig. 1e, f).

### Inhibition of STAT3 hindered the protective effect of daily PTH injections on alveolar bone mass in vivo

**Fig. 2:**
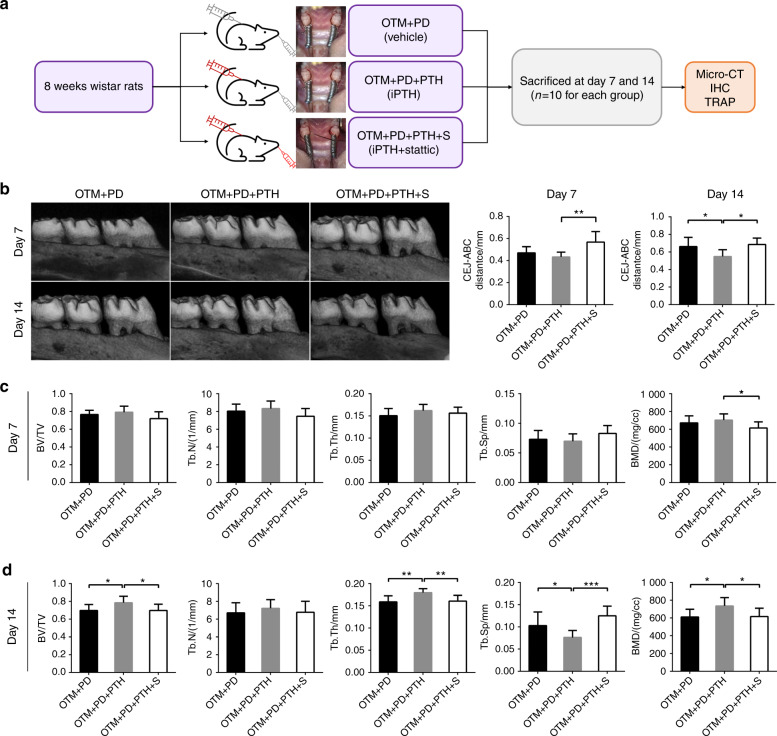
Daily systemic injection of PTH ameliorated alveolar bone loss in the OTM + PD group, which could be reversed by local stattic injection. **a** A flowchart of the in vivo study Part II. **b** Buccal images of specimens showing the vertical bone height in the OTM + PD, OTM + PD + PTH and OTM + PD + PTH + S groups, and quantitative analysis of the CEJ-ABC distance. **c**, **d** BV/TV, Tb. N, Tb. Th, Tb. Sp and BMD in each group at days 7 and 14, respectively. *P < 0.05, **P < 0.01, ***P < 0.001 by one-way ANOVA with Tukey’s post hoc test

Whether iPTH could ameliorate alveolar bone loss in the OTM + PD model and the role of STAT3 during this process were further investigated (Fig. 2a). On day 7, no significant difference was found in the CEJ-ABC distance or trabecular morphometry between the OTM + PD and OTM + PD + PTH groups (Fig. 2b, c). On day 14, compared with the OTM + PD group, the OTM + PD + PTH group showed a significant reduction in the CEJ-ABC distance and Tb. Sp, and notable elevation in the BV/TV, Tb. Th and BMD (Fig. 2b–d).

On day 7, the OTM + PD + PTH + S group showed a significant increase in the CEJ-ABC distance and a significant decrease in the BMD compared with the OTM + PD + PTH group (Fig. 2b, c). On day 14, the CEJ-ABC distance and Tb. Sp in the OTM + PD + PTH + S group were also significantly greater than those in the OTM + PD + PTH group, with marked decreases in the BV/TV, Tb. Th and BMD; these results indicated that the protective effect of iPTH was reversed by stattic in the OTM + PD + PTH + S group. No variation in the Tb. N was observed (Fig. 2b–d).

**Fig. 3:**
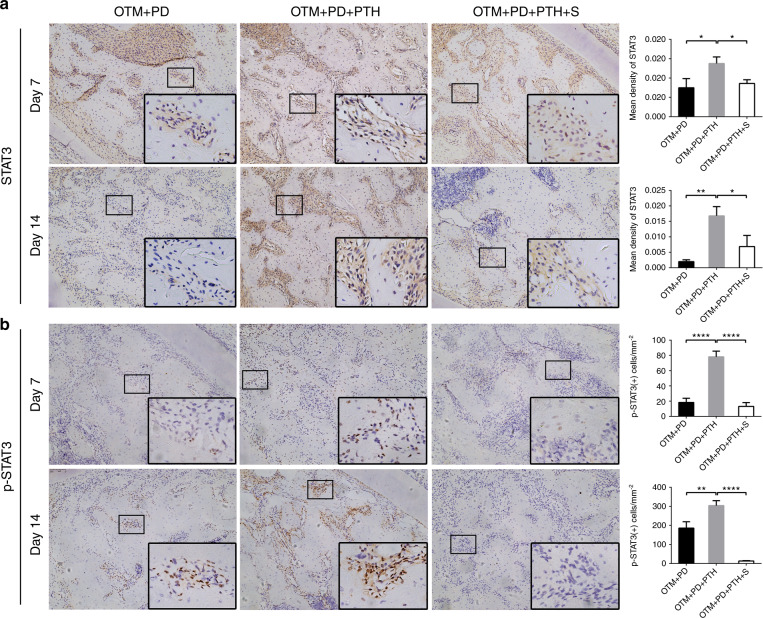
Daily systemic injection of PTH activated STAT3 in the alveolar bone, which could be reversed by local stattic injection. Representative images of immunohistochemical staining of STAT3 (**a**) and p-STAT3 (Tyr705) (**b**) in the OTM + PD, OTM + PD + PTH and OTM + PD + PTH + S groups, and relative quantitative analysis at days 7 and 14. *P < 0.05, **P < 0.01, ***P < 0.001, ****P < 0.000 1 by one-way ANOVA with Tukey’s post hoc test

The activation of STAT3 was also evaluated (Fig. 3a, b). Immunohistochemical staining showed that compared with the OTM + PD group, the OTM + PD + PTH group exhibited increased expression of STAT3 and numbers of p-STAT3 (Tyr705)-positive cells in the alveolar bone on days 7 and 14 after iPTH. Compared with the OTM + PD + PTH group, the OTM + PD + PTH + S group exhibited significantly decreased expression of STAT3 and numbers of p-STAT3 (Tyr705)-positive cells on day 7 and 14.

### Daily PTH injections promoted the formation of alveolar bone via the activation of STAT3 in vivo

**Fig. 4:**
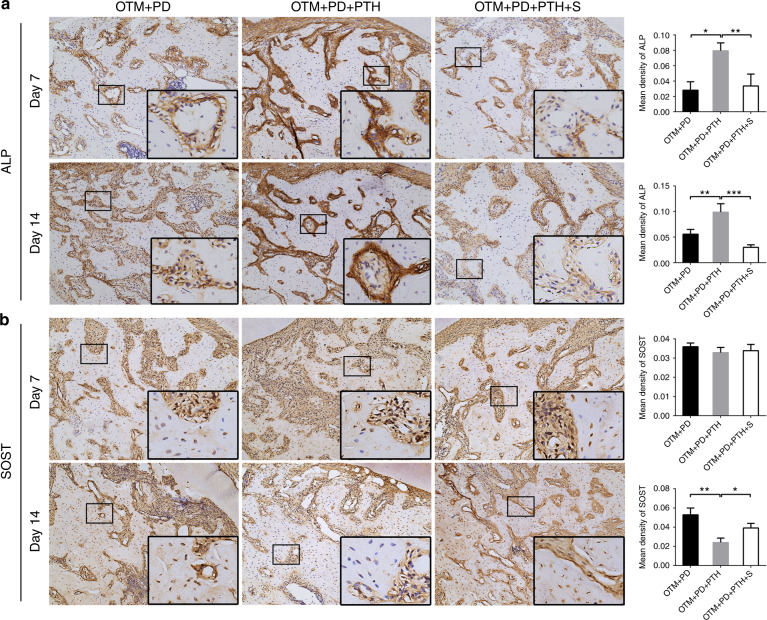
Daily systemic injection of PTH elevated the ALP levels and reduced the SOST levels in the alveolar bone, which could be reversed by local stattic injection. Representative images of immunohistochemical staining of ALP (**a**) and SOST (**b**) in the OTM + PD, OTM + PD + PTH and OTM + PD + PTH + S groups, and relative quantitative analysis at days 7 and 14. *P < 0.05, **P < 0.01, ***P < 0.001, ****P < 0.000 1 by one-way ANOVA with Tukey’s post hoc test

The immunohistochemistry results showed that iPTH significantly increased alkaline phosphatase (ALP) expression in alveolar bone on day 7 and 14, whereas stattic decreased this effect of iPTH (Fig. 4a). The expression of sclerostin (SOST), a negative regulator of bone formation, was also measured. On day 7, iPTH and stattic had no effect on the expression of SOST; on day 14, iPTH decreased the expression of SOST, whereas stattic increased the expression of SOST (Fig. 4b).

### Daily PTH injections inhibited the resorption of alveolar bone via the activation of STAT3 in vivo

**Fig. 5:**
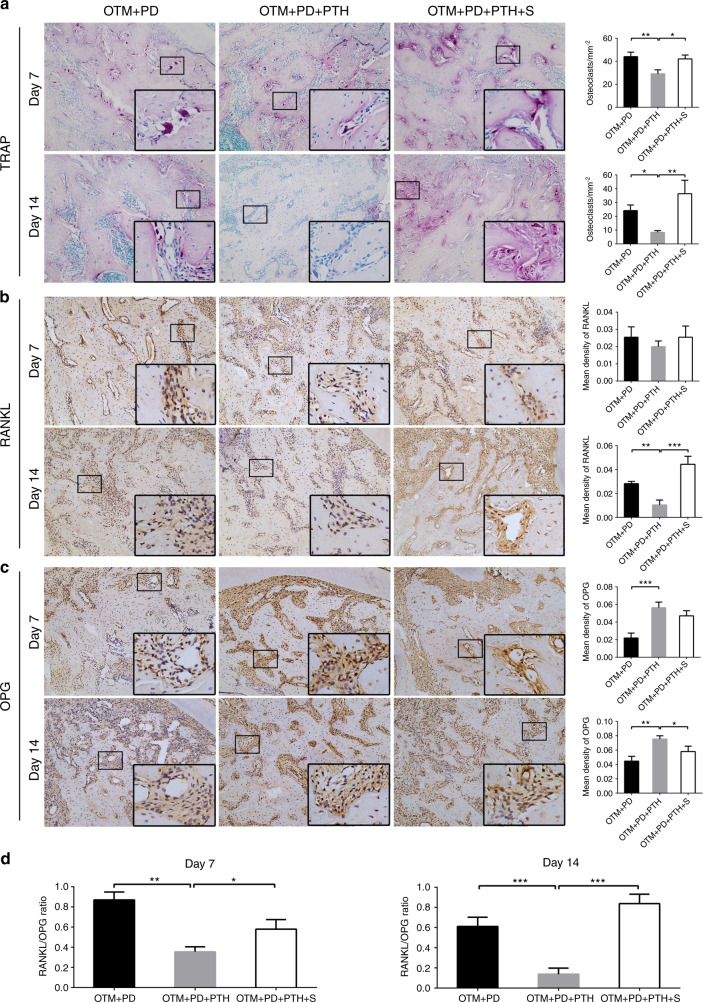
Daily systemic injection of PTH decreased osteoclast numbers and RANKL levels, and increased OPG levels, which could be reversed by local stattic injection. Representative images of TRAP staining (**a**), immunohistochemical staining of RANKL (**b**) and OPG (**c**) in the OTM + PD, OTM + PD + PTH and OTM + PD + PTH + S groups, and relative quantitative analysis at days 7 and 14. **d** Comparison of the RANKL/OPG ratio in each group. *P < 0.05, **P < 0.01, ***P < 0.001, ****P < 0.000 1 by one-way ANOVA with Tukey’s post hoc test

Tartrate-resistant acid phosphatase (TRAP) staining showed that iPTH significantly reduced the number of TRAP-positive multinucleated osteoclasts per unit area on days 7 and 14, while stattic increased the number of osteoclasts (Fig. 5a).

The expression of receptor activator of nuclear factor-κB ligand (RANKL) and the osteoprotegerin (OPG) system was also detected. On day 14, iPTH decreased the expression of RANKL, whereas stattic upregulated its expression. No significant change in the RANKL levels were observed on day 7 (Fig. 5b). IPTH significantly increased the OPG levels on day 7 and 14 (Fig. 5c). Stattic did not affect the OPG levels on day 7 but significantly decreased the OPG levels on day 14 (Fig. 5c). IPTH decreased the ratio of RANKL/OPG, whereas stattic significantly reversed this effect of iPTH on days 7 and 14. The change in the RANKL/OPG ratio in each group was basically consistent with the variation in the osteoclast numbers (Fig. 5d).

### IPTH promoted the osteogenesis of osteoblasts via the activation of STAT3 in vitro

**Fig. 6:**
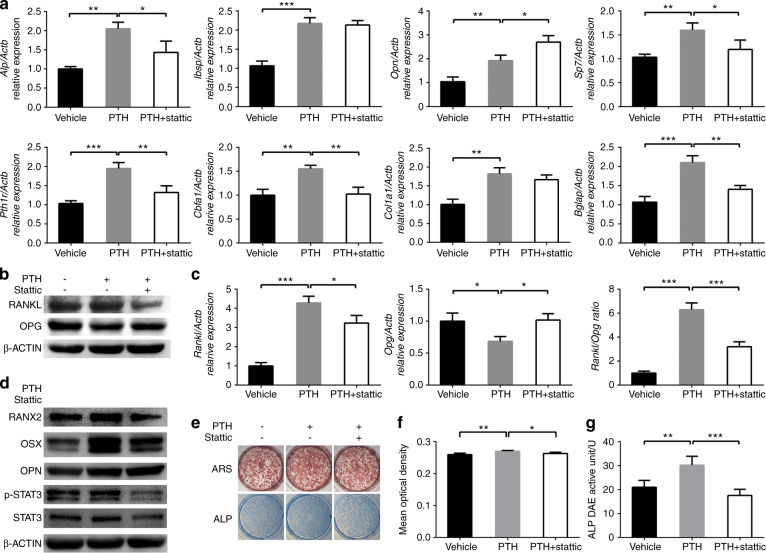
IPTH promoted osteogenesis in vitro, which could be reduced by stattic. **a** *Alp*, *Ibsp*, *Opn*, *Sp7*, *Pth1r*, *Cbfa1*, *Col1a1* and *Bglap* expression in each group. **b** Protein levels of RANKL and OPG. **c** RANKL and OPG mRNA levels and the RANKL/OPG ratio. **d** Protein levels of representative osteoblastic markers and p-STAT3 (Tyr705) and STAT3. **e** Alizarin red S staining and ALP staining. **f** Quantitative analysis of Alizarin red S staining. **g** Quantitative analysis of ALP. *P < 0.05, **P < 0.01, ***P < 0.001, ****P < 0.000 1 by one-way ANOVA with Tukey’s post hoc test

The effect of stattic on osteoblasts was first verified in vitro. As expected, the western blotting results showed that the STAT3 and p-STAT3 (Tyr705) levels in the osteoblasts in the PTH group were notably higher than those in the osteoblasts in the blank group, whereas the levels of both proteins were downregulated after coincubation with PTH and stattic compared with incubation with PTH alone after 3 days of iPTH treatment (Fig. 6d).

Then, the effect of iPTH on osteoblasts and the role of STAT3 in this process were investigated. At the end of the 3-day iPTH treatment, the expression levels of osteoblastic differentiation markers, including *Alp*, *Ibsp*, *Opn*, *Sp7*, *Pth1r*, *Cbfa1*, *Col1a1* and *Bglap*, were examined by quantitative real-time PCR (RT-qPCR). The expression levels of these genes were significantly increased after iPTH administration. Compared with PTH treatment alone, cotreatment with PTH and stattic significantly decreased the expression of *Alp*, *Cbfa1*, *Pth1r*, *Sp7* and *Bglap*, and increased the expression of *Opn*, whereas *Col1a1* and *Ibsp* expression did not significantly change (Fig. 6a). The protein levels of runt-related transcription factor 2 (RUNX2), osterix (OSX) and osteopontin (OPN) were measured through western blotting and the results were consistent with the gene expression results (Fig. 6d). Both the RT-qPCR and western blotting results were normalized to β-actin expression.

Consistently, iPTH treatment significantly enhanced ALP activity, as indicated by cytochemical staining and quantitative assays (Fig. 6e–g). Alizarin red S staining showed that mineralization ability was promoted by iPTH. Stattic, on the other hand, reversed the effects of PTH described above (Fig. 6e, f).

### IPTH elevated the RANKL/OPG ratio in osteoblasts via STAT3 activation in vitro

To investigate the effect of iPTH on osteoclastogenesis through its target cell, namely osteoblasts, the gene and protein levels of RANKL and OPG were measured after 3 days of iPTH treatment. IPTH treatment upregulated the expression of RANKL and downregulated the levels of OPG in osteoblasts, resulting in an increased RANKL/OPG ratio, whereas stattic reversed the effect of iPTH on the RANKL and OPG levels, and the RANKL/OPG ratio, as indicated by both RT-qPCR and western blotting (Fig. 6b, c).

### STAT3 was involved in the PTH-induced activation of Wnt/β-catenin signalling

**Fig. 7:**
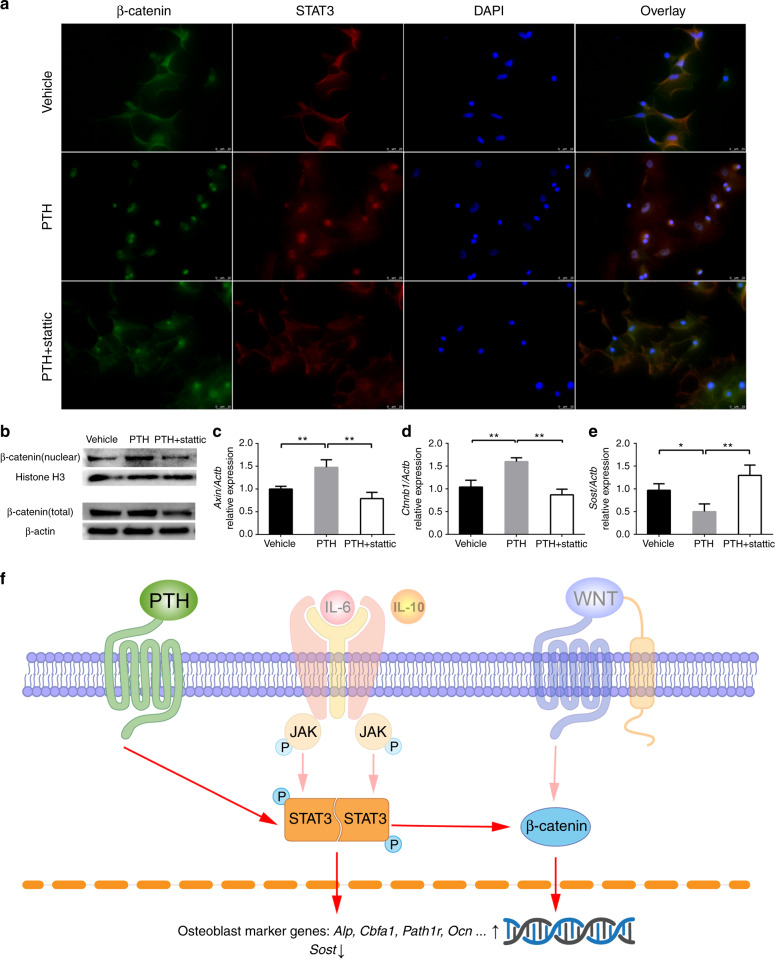
IPTH activated Wnt/β-catenin signalling, while inhibition of STAT3 activation suppressed this effect. **a** Colocalization of β-catenin and STAT3 by immunofluorescence. **b** β-catenin levels in nuclear fractions (active form) and whole cells. **c** *Axin2* expression level. **d** *Ctnnb1* expression level. **e** *Sost* expression level. **f** A diagram showing the hypothesis that PTH regulates STAT3 and Wnt/β-catenin signalling. *P < 0.05, **P < 0.01 by one-way ANOVA with Tukey’s post hoc test

The degree of Wnt/β-catenin signalling activation was determined in each group. Immunofluorescence analysis of the colocalization of STAT3 and β-catenin indicated that PTH caused both proteins to translocate from the cytoplasm to the nucleus, which indicated that both signalling pathways were activated. PTH plus stattic treatment prevented both proteins from entering the nucleus, suggesting that the inactivation of STAT3 may inhibit Wnt/β-catenin signalling (Fig. 7a). This effect was also verified by western blotting analysis of nuclear β-catenin (Fig. 7b). In addition, the expression level of *Axin2*, a target gene of the Wnt/β-catenin signalling pathway, was consistent with the activation level of this signalling pathway in each group (Fig. 7c). The total level of β-catenin was also measured. Both RT-qPCR and western blotting analyses showed that the expression of β-catenin was increased by PTH and decreased by stattic (Fig. 7b–d). IPTH downregulated the expression of *Sost*, whereas iPTH and stattic cotreatment upregulated the expression of *Sost* (Fig. 7e). All these results indicated that increased activation of Wnt/β-catenin signalling by iPTH was related to STAT3 (Fig. 7f).

## Discussion

In this study, a ligature was used to induce PD, which caused a reduction in alveolar bone height and trabecular bone density, breakdown of gingiva, pyorrhoea in gingival sulcus and bleeding on probing on days 7 and 14; these results indicated that the animal model of PD had been successfully established.^12^ OTM exacerbated the alveolar bone loss induced by PD, as indicated by the increased CEJ-ABC distance and more porotic trabecular bone, which is in agreement with previous clinical and animal studies in rats.^5,7,41–45^

After 14 days of iPTH treatment, compared with the OTM + PD group, the OTM + PD + PTH group showed significantly reduced alveolar bone resorption and improved trabecular bone parameters. However, this effect was not obvious on day 7, which suggested delayed protection of alveolar bone mass by iPTH. A similar protective effect of iPTH on alveolar bone with active inflammation was also observed in several studies of murine models. IPTH has been found to prevent the bone loss induced by PD,^15^ bacterial periapical inflammation,^18^ arthritis^46^ and tooth extraction,^47^ and significant results have been observed as early as day 7.^47^ In addition, inflammatory cell infiltration was inhibited and bone loss was reduced by iPTH in the studies mentioned above. These studies suggest that iPTH may also have an anti-inflammatory effect in addition to its regeneration-promoting effect and these mechanisms need to be elucidated by further research.

Our results also suggested that the protective effect of iPTH on alveolar bone may be mediated by the activation of STAT3. Local injection of stattic significantly promoted damage to alveolar bone crest height on day 7 and day 14 and affected trabecular morphometry mainly on day 14. STAT3 is an important mediator of inflammation. Lipopolysaccharide (LPS) decreases STAT3 phosphorylation and the LPS-induced production of RANKL and proinflammatory cytokines, such as IL-6, relies on STAT3 phosphorylation in periodontal ligament cells.^48^ However, STAT3 activation is also indispensable for anti-inflammatory processes, such as IL-10 receptor signalling.^49,50^ Due to the mitigating effect of iPTH on inflammatory bone loss observed in this study, we inferred that STAT3 may play an anti-inflammatory role in the mechanism by which iPTH preserves bone mass.

ALP activity was measured as an indicator of bone formation. On day 7 and 14, the expression of ALP in alveolar bone was significantly upregulated by iPTH, indicating that osteogenic activity was enhanced. The results of Bashutski et al.^19^ also showed that the plasma level of ALP in patients with severe chronic PD was significantly increased after iPTH treatment. In addition to the serum levels of ALP, the serum levels of vitamin D, osteocalcin and C-telopeptide of type I collagen were also increased by iPTH treatment.^19,51^ In this study, the inactivation of STAT3 decreased the iPTH-induced upregulation of ALP. Previous studies showed that STAT3 inactivation in mesenchymal stem cells led to reduced osteogenic differentiation and decreased ALP expression, whereas activated STAT3 overexpression promoted osteogenic differentiation and ALP expression.^52^ These findings are consistent with our results. The present in vitro experiment also supported the in vivo results that iPTH activated STAT3 and, as a result, promoted the expression of osteoblastic genes, including *Alp*, *Cbfa1*, *Pth1r*, *Sp7* and *Bglap*, and osteogenic function. Although stattic reversed the effect of iPTH on increasing the expression of most osteoblastic genes, we found that stattic had no effect on the regulation of the expression of *Col1a1* and *Ibsp*. Moreover, stattic even showed a significant promoting effect on *Opn* at both the mRNA and protein levels. As a marker of mature osteoblasts, OPN is a negative regulator of bone formation;^53^ thus, this result actually indicated less active bone formation when STAT3 was inhibited and did not contradict the rest of our results.

PTH regulates osteoclast activities predominantly through cells of the osteoblastic lineage by changing RANKL and OPG expression and the RANKL/OPG ratio.^54^ Our animal study showed that iPTH decreased the number of osteoclasts in alveolar bone and that the RANKL levels were decreased and the OPG levels were increased; these effects could be reversed by the inhibition of STAT3. The effect of iPTH on RANKL and OPG expression in vitro, however, conflicts with the effect observed in vivo. This contradiction was also observed in previous studies. IPTH was reported to elevate RANKL expression in osteoblastic cells in vitro,^55,56^ whereas in vivo research indicated that in diabetic rats with PD, iPTH decreased the RANKL/OPG ratio and exerted an anabolic effect on inflammatory alveolar bone.^17^ The different effects of iPTH on osteoclasts may be caused by the different cell responses to iPTH under normal and inflammatory conditions, as it was difficult to absolutely mimic the in vivo inflammatory environment. In addition, the regulation of osteoclasts by iPTH may involve various cell types, including lymphocytes and osteocytes, which are also sources of RANKL and may exert anti-inflammatory effects in response to PTH and proinflammatory factors.^34,57^ Future studies are needed to further elucidate the regulatory mechanisms of iPTH in osteoclasts.

Wnt/β-catenin signalling plays a critical role in bone formation and β-catenin is the core signalling transducer of this pathway. After the Wnt/β-catenin pathway is activated, β-catenin is stabilized, leading to its accumulation in the cytoplasm and resulting in its translocation to the nucleus to regulate gene expression. We assumed that STAT3 might regulate bone formation by affecting Wnt/β-catenin signalling. Our study showed that STAT3 inactivation led to decreased Wnt/β-catenin activation in osteoblasts after PTH treatment, as well as to decreased β-catenin levels in the nucleus and reduced expression of *Axin2*. The results described above suggested an effect of PTH on Wnt/β-catenin signalling activation and STAT3-associated nuclear translocation of β-catenin. Consistent with previous research,^58–60^ iPTH also inhibited SOST expression, and we discovered that this downregulation of *Sost* expression depended on STAT3 activation. Further experiments are needed to elucidate the mechanisms by which STAT3 regulates the Wnt/β-catenin pathway in bone metabolism.

In conclusion, the results in this study suggest that systemic iPTH treatment decreases alveolar bone loss during OTM in rats with PD in a STAT3-dependent manner that involves STAT3/β-catenin interactions.

## Materials and methods

### Animal models

The study was approved by the Research Ethics Committee of State Key Laboratory of Oral Diseases, West China Hospital of Stomatology, Sichuan University (WCHSIRB-D-2017–236).

Sixty 8-week-old male Wistar rats (Experimental Animal Center, Sichuan University, China) were randomly divided into six groups (*n* = 10 in each group): the blank group, the PD group, and the OTM + PD group, and each group included subgroups analysed at two time points (day 7 and 14). To induce PD, a 0.2 mm stainless steel wire was ligated onto the cervix of the maxillary first molars in the PD group. To induce OTM + PD, NiTi coil springs were calibrated to 40 g with a dynamometer and ligated with a 0.2 mm stainless steel wire onto the cervix of the bilateral maxillary first molars, and the ipsilateral incisors were used as anchor teeth. The blank group was left untreated (Fig. 1a). The devices were evaluated daily and no breakage or failure was observed throughout the study.

Another 60 male Wistar rats were randomly divided into six groups (*n* = 10 in each group): the OTM + PD group, the OTM + PD with iPTH treatment group (OTM + PD + PTH), and the OTM + PD with iPTH and local stattic injection group (OTM + PD + PTH + S), and each group included subgroups analysed at time points (day 7 and 14) (Fig. 2a).

For iPTH administration, human PTH (1–34) (Bachem, USA) diluted in normal saline was subcutaneously injected on the back (40 μg·kg^−1^ body weight) of the rats at the same time every day. Stattic (Selleck, USA), a small molecule inhibitor of STAT3, was used to locally inhibit STAT3. Stattic was dissolved in dimethyl sulfoxide (DMSO) and diluted in normal saline (with a final DMSO concentration of 0.106%) to achieve a final concentration of 50 μmol·L^−1^. Then, 10 μL stattic working solution was injected beneath the mucoperiosteum on the palatal side of the maxillary first molars every 2 days. The groups not administered drugs were injected with an equal volume of the corresponding vehicle in the same manner.

Ten rats in each group were sacrificed by an overdose of anaesthesia 24 h after the last PTH injection on day 7 and 14. The maxillae were collected and fixed in 4% paraformaldehyde for 24 hours for the subsequent experiments.

### Micro-CT scanning and analysis

Specimens were scanned with an X-ray tube potential of 70 kVp, current of 200 mA and voxel size of 10 μm. To assess the height of alveolar bone loss, the distance between the CEJ and ABC on the buccal side of the maxillary first molar was measured with three-dimensional images (Fig. 1b). To analyse the trabecular morphometry, the BV/TV, Tb. N, Tb. Th, Tb. Sp and BMD were measured. The region of interest was defined as the intraradicular area of the maxillary first molar (Fig. 1c) with a thickness of 500 μm.

### Immunohistochemistry

Maxillae were decalcified in 10% ethylenediaminetetraacetic acid solution, bedded in paraffin and sectioned into 5 μm-thick sagittal slices. After being deparaffinized and hydrated, the sections were treated with 0.1% trypsin for 30 min at 37 °C for antigen retrieval, then with 3% hydroperoxide for peroxidase inactivation and then with 5% bovine serum albumin (BSA) for 1 h at room temperature for blocking. Rabbit primary antibodies against STAT3, ALP, RANKL, OPG and SOST (Huabio, China) were dissolved in 1% BSA, added to the sections and incubated at 4 °C overnight. The SP link detection kits (Biotin-Streptavidin HRP Detection Systems) and a DAB colouration kit (ZSGB-BIO, China) were used for the detection of immunoactivity. Finally, haematoxylin was used to stain the nuclei. Images were quantified with Image-Pro Plus 6.0 (Media Cybernetics, USA).

### TRAP staining

TRAP staining of the paraffin sections was performed using the TRAP/ALP stain kit (Fujifilm WAKO, Japan) according to the manufacturer’s instructions. Images were quantified with Image-Pro Plus 6.0 (Media Cybernetics, USA).

### Cell culture and treatments

Primary bone marrow mesenchymal stem cells (BMSCs) were obtained from the tibiae and femurs of 2-week-old male Sprague–Dawley rats (Experimental Animal Center, Sichuan University, China) and cultured using a published protocol.^61^ The BMSCs were cultured in alpha-minimum essential medium (Gibco, USA) with 10% fetal bovine serum (Gibco, USA), 100 U·mL^−1^ penicillin G and 100 μg·mL^−1^ streptomycin (HyClone, USA) in a humidified chamber (37 °C and 5% CO_2_). After reaching 90% confluence, the cells were subcultured or seeded into plates. To induce osteoblastic differentiation and mineralization, proliferation medium was supplemented with 2  nmol·L^-1^ β-glycerophosphoric disodium, 100 nmol·L^−1^ dexamethasone and 50 μg·mL^−1^ ascorbic acid to produce osteogenic medium, and the medium was refreshed every 3 days. Osteoblasts were obtained by inducing the mineralization of BMSCs for 14 days, at which point, mineralization nodules appeared.

The osteoblasts were then divided into the vehicle group, PTH group and PTH + stattic group. For iPTH treatment, the proliferation medium in the PTH group and vehicle group was replaced with osteogenic medium containing 100 ng·mL^−1^ human PTH (1–34) (Bachem, USA) or vehicle (1% acetic acid), respectively, and incubated for the first 6 h; then, the medium was changed to osteogenic medium without PTH or vehicle and incubated for the following 18 h, completing one cycle, as previously described.^62^ To examine the effect of PTH on STAT3 inactivation, PTH and stattic (5 μmol·L^−1^) were added to the medium and incubated for the first 6 h, and the cells were incubated in medium containing only stattic for the following 18 h in each cycle. The total iPTH treatment lasted for three cycles.

### ALP staining and quantitative assay

At the end of the iPTH treatment, the ALP activity in osteoblasts was measured. A BCIP/NBT ALP colour development kit (Beyotime, China) was used for staining and an ALP assay kit (Beyotime, China) was used for the quantitative assay according to the manufacturer’s instructions.

### Alizarin red S staining and quantification

After iPTH treatment, osteoblasts were cultured in osteogenic medium until day 21 to detect calcium deposits. Then, the cells were fixed in 4% paraformaldehyde for 30 min, stained with 1% alizarin red S dye (pH 4.2) (Solarbio, China) for 5 min, and washed with distilled water before stereomicroscopy analysis. The greyscale value of each stained well was measured with Image-Pro Plus 6.0.

### Quantitative real-time PCR

At the end of the iPTH treatment, the total RNA was extracted with TRIzol as previously described^63^ and cDNA was generated using a RevertAid First-Strand cDNA Synthesis Kit (Thermo Fisher Scientific, USA). RT-qPCR was performed using TB Green^®^ Premix Ex Taq™ II (TaKaRa, Japan) on a QuantStudio 3 Real-Time PCR System (Thermo Fisher Scientific, USA).

### Western blotting

At the end of the iPTH treatment, the total protein was extracted using the Total Protein Extraction Kit (Signalway Antibody, USA), the Nuclear protein was extracted using a Nucleoprotein Extraction Kit (Sangon Biotech, China). Western blotting was conducted as previously described.^62^ Briefly, the protein concentration was determined using a BCA Protein Assay Kit (Beyotime, China). After denaturation, the samples were loaded onto SDS-polyacrylamide gel electrophoresis gels for electrophoresis and transferred to polyvinylidene difluoride membranes. The membranes were blocked with 5% skim milk or 5% BSA and incubated with primary antibodies against β-actin (Huabio, China), STAT3 (Huabio, China), phospho-STAT3 (Tyr705) (Cell Signaling Technology, USA), RUNX2 (Huabio, China), OSX (Abcam, UK), ALP (Huabio, China), OPN (Huabio, China), β-catenin (Huabio, China), RANKL (Abcam, UK) and OPG (Huabio, China) at 4 °C overnight, and rabbit and mouse secondary antibodies (Signalway Antibody, USA). The bands were visualized with a ChemiDoc Touch Imaging System (Bio-Rad, USA) and quantified with Image-Pro Plus 6.0.

### Immunofluorescence

To colocalize STAT3 and β-catenin, double-label immunofluorescence was conducted. Six hours after PTH treatment or PTH and stattic cotreatment, the cells were fixed with 4% paraformaldehyde for 30 min, permeabilized with 0.5% Triton X-100 for 20 min, blocked with 5% BSA for 30 min, and incubated with rabbit primary antibodies against STAT3 (Huabio, China) and mouse primary antibodies against β-catenin (Huabio, China) at 4 °C overnight. The cells were then stained with rhodamine-labelled anti-rabbit secondary antibodies and fluorescein isothiocyanate-labelled anti-mouse secondary antibodies (Huabio, China) for 1 h at room temperature and counterstained with 4′,6-diamidino-2-phenylindole for 10 min. Images were captured with a fluorescence microscope (Leica, Germany).

### Statistical analysis

The data are presented as the mean ± SD. One-way analysis of variance with Tukey’s post hoc test for multiple comparisons was performed in SPSS 13.0 (IBM, USA) for statistical comparisons. A *P*-value < 0.05 was considered statistically significant.
